# Anti-Inflammatory Therapies for Atopic Dermatitis: A New Era in Targeted Treatment

**DOI:** 10.3390/jcm14145053

**Published:** 2025-07-16

**Authors:** Karol Biliński, Katarzyna Rakoczy, Anna Karwowska, Oliwia Cichy, Aleksandra Wojno, Agata Wojno, Julita Kulbacka, Małgorzata Ponikowska

**Affiliations:** 1 Faculty of Medicine, Wroclaw Medical University, L. Pasteura 1, 50-367 Wroclaw, Poland; karol.bilinski@student.umw.edu.pl (K.B.); katarzyna.rakoczy@student.umw.edu.pl (K.R.); anna.karwowska@student.umw.edu.pl (A.K.); oliwia.cichy@student.umw.edu.pl (O.C.); aleksandra.wojno@student.umw.edu.pl (A.W.); agata.wojno@student.umw.edu.pl (A.W.); 2 Student Research Group No. K148, Faculty of Pharmacy, Wroclaw Medical University, Borowska 211A, 50-556 Wroclaw, Poland; 3 Department of Molecular and Cellular Biology, Faculty of Pharmacy, Wroclaw Medical University, Borowska 211A, 50-556 Wroclaw, Poland; 4 Department of Immunology and Bioelectrochemistry, State Research Institute Centre for Innovative Medicine, Santariškiu˛ g. 5, LT-08406 Vilnius, Lithuania; 5 University Center of General and Oncological Dermatology, Wroclaw Medical University, Borowska 213, 50-556 Wroclaw, Poland

**Keywords:** atopic dermatitis, biological therapy, cytokines, targeted treatment, biomarkers, dupilumab

## Abstract

Atopic dermatitis (AD) is a chronic, relapsing inflammatory skin condition characterized by intense pruritus and a significant impact on a patient’s quality of life. Despite advancements in understanding AD pathophysiology, there remains a critical need for innovative therapeutic options to better manage this debilitating disease. This review focuses on the evolving landscape of biological therapies for AD, offering insights into their role, mechanisms of action, and potential to revolutionize patient care. In this review, we explore the underlying immunological mechanisms of AD, particularly the role of cytokines and immune pathways implicated in the disease, and how targeted biological therapies modulate these pathways. Current FDA- and EMA-approved biologics, such as Dupilumab, are also discussed in terms of their mechanisms of action, efficacy, and safety. Additionally, we compare their effectiveness, highlighting the benefits and limitations observed in clinical practice. Emerging biological therapies currently under development offer new hope, with innovative targets like IL-13, IL-31, and thymic stromal lymphopoietin (TSLP) representing promising avenues for intervention. We also delve into personalized medicine, emphasizing the importance of biomarkers for predicting treatment response and stratifying AD patients to optimize therapeutic outcomes. Moreover, the synergistic potential of combining biologics with traditional therapies is reviewed, along with a discussion of the challenges involved, including safety, long-term efficacy, and patient adherence. We address the future direction of AD treatment, including microbiome-targeting biologics and the development of next-generation immune modulators. We highlight a new era of targeted treatment possibilities for this complex condition.

## 1. Introduction

Atopic dermatitis (AD) is one of the most common inflammatory diseases, with close to 204.05 million people affected globally [1]. The prevalence of AD varies across different regions of the world, with a generally increasing trend [2]. Sixty percent of children with AD show symptoms within the first year of life, and 90% develop symptoms by the age of five [3]. In the pediatric population, the prevalence reaches approximately 20% [4], while among adults, it stands at 3.5% in Europe [5]. The most common symptoms of the disease include persistent and intense pruritus and inflamed lesions on the skin, which significantly impact various aspects of daily life, such as quality of sleep and work productivity, leading to increased stress and reduced general happiness [6,7]. Moreover, the clinical presentation of the disease varies across different age groups affected by AD. In children under the age of two, the disease often manifests as papules and vesicles with crusting that are primarily localized to the head and neck. In contrast, adults predominantly exhibit lesions in the flexural areas and distal extremities. Characteristic lesions for this age include symmetric, dry, scaly papules and plaques, with lichenification occurring frequently as a secondary change [8].

Due to its chronic nature, AD is associated with substantial social and economic problems for the affected population. Studies indicate that patients with AD experience nine flares per year, each lasting 15 days, totaling 136 days in flare annually. AD flares disrupt sleep, with severe cases experiencing significantly more sleep disturbance (14.6 nights per flare, 162 nights per year). An analysis of AD’s impact on adult work performance and absenteeism reveals that lost productivity costs the European Union over EUR 2 billion annually [9]. In 2024, the FDA approved several new biological treatments for AD, targeting innovative pathways. These include nemolizumab, which inhibits IL-31; roflumilast, a selective inhibitor of phosphodiesterase-4; tapinarof, which decreases the expression of IL-17 and IL-22; and lebrikizumab, a monoclonal antibody targeting IL-13. Recent advances have broadened therapeutic options for AD patients, creating opportunities for optimized and targeted therapy [10].

However, despite the increasing number of therapeutic options, there remains a lack of comprehensive, up-to-date narrative reviews that integrate recent clinical trial data, compare emerging agents, and discuss their positioning within treatment algorithms for AD. In particular, clinicians and researchers need clear guidance on the mechanisms of action, efficacy, safety profiles, and practical considerations associated with both established and investigational biologics and small-molecule inhibitors.

Therefore, the aim of this narrative review is to provide an updated synthesis of current and emerging anti-inflammatory therapies for AD, with a focus on biological agents. We seek to contextualize recent advances within the broader pathophysiological framework of AD, critically evaluate clinical evidence supporting various treatment options, and discuss future directions for personalized and combination therapies. By highlighting these aspects, we aim to provide clinicians and researchers with a practical and insightful resource to support informed decision-making in the evolving management of AD.

## 2. Methods

This review was conducted as a narrative overview of current and emerging biologic and targeted therapies for AD. Relevant articles were identified by performing a structured search of the PubMed and Google Scholar databases. The search included publications from January 2018 to May 2025 to ensure the inclusion of recent clinical trial data and emerging therapeutic agents.

The following keywords and their combinations were used: “atopic dermatitis,” “biological therapy,” “biologics,” “cytokines,” “dupilumab,” “tralokinumab,” “JAK inhibitors,” “combination therapy,” “immunosuppression,” and “targeted therapy.”

Inclusion criteria were as follows:

- peer-reviewed articles in English,
- studies involving human participants (adults and/or children),
- original clinical trials, meta-analyses, or systematic reviews, and
- papers discussing mechanisms of action, efficacy, safety, or treatment algorithms for biologic agents in AD.

Exclusion criteria included

- non-peer-reviewed articles,
- animal-only studies, and
- case reports or conference abstracts without full data.

Reference lists of the included articles were also manually screened to identify additional relevant publications. Although the main focus was placed on studies from the last seven years, older sources were included when they provided an essential background or historical context for the development of current treatment paradigms.

## 3. Pathophysiology of Atopic Dermatitis

In the pathophysiology of atopic dermatitis (AD), multiple variables interact in a complex manner, frequently triggering and exacerbating one another. These factors include genetic predisposition, immune system dysregulation, skin barrier dysfunction, microbiome alterations, and environmental triggers [11]. In the 2024 article by Wu et al., the existence of a positive feedback loop that facilitates the progression of epidermal barrier dysfunction is stated [12]. The predominance of shorter lipid chains and the decrease in lipid content promote microbiome disturbance and the activation of adaptive immunity [12]. Local dysregulation of the immune system and activation of Th2 lymphocytes further impair epidermal barrier dysfunction, promoting the colonization of the skin by S. aureus and other opportunistic bacteria [11]. Emerging evidence also underscores the role of fungal dysbiosis, particularly involving *Malassezia* and *Candida* species, in exacerbating the immune response and disease severity in AD. These insights highlight the potential of novel targeted therapies, including antifungal strategies and mycobiome-modulating treatments, to enhance disease management in the future [13].

### 3.1. Genetic Factors

Research has shown that a family history of atopic disease is a strong predictive factor for the development of AD, with an average heritability of 75% [11]. The principal protein essential for epidermal barrier function is filaggrin, which is present in keratinocytes within the stratum granulosum [14]. Several factors can contribute to filaggrin deficiency, with FLG mutations located on chromosome 1q21 being the most common. This mutation has been proven to be the strongest genetic risk factor for the development of AD. Notably, FLG gene variants can interact with environmental factors, including hard water and phthalates, whose exposure enhances AD risk [15]. Although FLG is the most widely described, there are other genetic susceptibilities involved in AD pathogenesis, such as OVOL1, CARD14, and KIF3A; however, precisely determining their role remains a challenge [15].

### 3.2. Inflammation Mechanism

Cytokines play a crucial role in the pathogenesis of AD. The involvement of Th2, Th17, and Th22 is considered; however, Th2 lymphocytes, particularly due to the production of IL-4 and IL-13, are pivotal in the process [16]. Cytokines also influence several subjective symptoms experienced by patients. IL-31 has been implicated in inducing pruritus and enhancing neurotransmission [17]. IL-22 plays a crucial role in skin homeostasis, providing communication between the immune system. It also influences keratocytes’ proliferation and contributes to epidermal hyperplasia [16,18]. However, the most important cytokines for AD pathophysiology appear to be IL-4 and IL-13. IL-4 influences all of the key aspects of AD: it induces pruritus by promoting the production of IL-31, contributes to skin dysbiosis through excessive collagen production that enhances S. aureus adhesion, and drives inflammation by activating dendritic cells and promoting the production of IgE [19]. Various cell types, such as Th2 lymphocytes, mast cells, and basophils, produce IL-13. IL-13 has been proven to reduce skin barrier integrity by downregulating the expression of filaggrin, loricrin, and desmoglein. Additionally, IL-13 is part of a positive feedback loop associated with skin dysbiosis, facilitating S. aureus adhesion [20]. It is essential to note that the described inflammatory mechanisms primarily pertain to the extrinsic subtype of AD, which is characterized by an elevated Th2 immune response and increased serum IgE levels. In contrast, the intrinsic subtype of AD, although less common, presents with clinical manifestations of AD but lacks a history of other atopic diseases and demonstrates negative type I hypersensitivity reactions to aeroallergens and food allergens. Patients with intrinsic AD typically exhibit normal total serum IgE concentrations and an absence of detectable allergen-specific IgE antibodies [21]. Patients with intrinsic AD often display a less pronounced Th2-skewing immune profile, characterized by higher expression of IFN-γ-producing Th1 cells, normal skin barrier function, and a lower frequency of FLG mutations compared to those with extrinsic AD [22,23]. In contrast, the extrinsic form of AD is associated with higher serum levels of CCL17/TRAC (thymus and activation-regulated chemokine), a chemokine that attracts Th2 cells, and more pronounced eosinophilic infiltration in skin lesions [22]. The overproduction of IFN-γ in intrinsic AD may contribute to the suppression of IgE synthesis, further differentiating this subtype immunologically from the extrinsic form [23,24]. These differences in the cytokine microenvironment underscore the heterogeneity of AD and highlight the importance of distinguishing between AD endotypes to enable more personalized and targeted therapeutic strategies.

### 3.3. Key Immunological Mechanisms and Therapeutic Targets in AD

The Janus kinase (JAK)–signal transducer and activator of transcription (STAT) pathway constitute a central intracellular signaling mechanism critically involved in the immunopathogenesis of AD. The JAK kinase family consists of four receptor-associated tyrosine kinases: JAK1, JAK2, JAK3, and tyrosine kinase 2 (TYK2), while the STAT family comprises seven transcription factors: STAT1, STAT2, STAT3, STAT4, STAT5A, STAT5B, and STAT6 [25]. In AD, IL-4, a cytokine pivotal for disease pathogenesis, binds to the type I IL-4 receptor (IL-4R), leading to the phosphorylation of JAK1 and JAK3. This, in turn, activates IL-4Rα and induces the phosphorylation of STAT6. Phosphorylated STAT6 dimerizes and translocates into the nucleus, where it functions as a transcription factor for IL-4-responsive genes involved in promoting Th2 polarization, IgE synthesis, and suppression of skin barrier proteins such as filaggrin. During the chronic phase of AD, cytokines derived from Th17 and Th22 cells—such as IL-17 and IL-22—contribute to sustained inflammation and epidermal hyperplasia via both JAK-STAT-dependent and independent pathways. While IL-17 primarily activates ACT1-TRAF6 complexes leading to NF-κB and MAPK signaling, IL-22 signals through JAK1 and TYK2, with downstream activation of STAT3 as the main transcriptional effector, further amplifying keratinocyte proliferation and tissue remodeling [25].

Thymic stromal lymphopoietin (TSLP) is an epithelial-derived cytokine whose expression is markedly elevated in AD and correlates with disease severity and skin barrier dysfunction [26]. Functionally, TSLP signals via a receptor complex engaging JAK1/JAK2 and predominantly activates STAT5, promoting maturation of dendritic cells and upregulation of OX40L, expressed on activated antigen-presenting cells. The co-stimulatory T-cell receptor OX40 is predominantly expressed in effector and regulatory T-cells. Engagement of the OX40–OX40L axis facilitates the expansion and survival of T-cells by inhibiting apoptosis and augmenting cytokine production, particularly by Th2 cells, thereby promoting the secretion of key for AD cytokines such as IL-4 and IL-13 [26,27,28].

## 4. Personalized Medicine in AD

The traditional therapeutic approach to AD has primarily focused on symptomatic treatment. However, in recent years, there has been growing interest in personalized medicine, which tailors therapeutic interventions to the individual characteristics of each patient. A critical component of this approach is the identification of biomarkers that can predict treatment responses, thereby facilitating the optimization of therapy [29]. Biomarkers are defined characteristics that serve as measurable indicators of biological states, reflecting interactions between biological systems and potential external threats, whether chemical, physical, or biological in origin [30]. According to the International Eczema Council (IEC), AD is a highly heterogeneous disease with at least three distinct phenotypes. Biomarkers play a crucial role in stratifying patients based on these phenotypes, which can enhance clinical management and promote better treatment adherence [31]. This stratification enables a more precise and individualized approach to treatment, targeting the specific pathological mechanisms underlying the disease in each phenotype.

Clinical studies have identified several potential subtypes of biomarkers for AD. However, none of these candidates have yet been adopted into routine clinical practice, as they have not achieved the necessary levels of validation and qualification [32]. For instance, IL-36γ, IL-1F9 have been proposed as markers to differentiate AD from psoriasis, as their expression is significantly elevated in the skin lesions of patients with psoriasis [33]. The development of biomarkers in AD is particularly aimed at predicting therapeutic response and monitoring disease progression, which are considered critical objectives [34]. Biomarkers in AD can be classified into various subtypes based on their intended clinical application. These include (1) biomarkers for assessing disease severity and monitoring activity, such as Th2- and Th22-related cytokines and chemokines [35]; (2) prognostic and screening biomarkers, such as mutations in the FLG gene [36]; (3) predictive biomarkers, including high serum levels of periostin and dipeptidyl peptidase-4 [37]; and (4) diagnostic biomarkers, such as nitric oxide synthase 2 (NOS2), though this particular biomarker has not yet been clinically validated [38]. These categories reflect the diverse roles that biomarkers can play in advancing personalized medicine for AD. However, further research and standardization are needed to translate these findings into routine clinical use.

The stratification of patients with AD relies on the identification of biomarkers capable of predicting responses to specific therapies. These biomarkers encompass both clinical and molecular parameters. For instance, elevated levels of IL-13 and IL-4 are characteristic of the Th2 phenotype, which predominates in AD. Patients exhibiting this phenotype may demonstrate enhanced therapeutic responses to drugs targeting the IL-4/IL-13 signaling pathways, such as dupilumab [39]. Traditionally, AD has been classified into intrinsic and extrinsic subtypes based on factors such as age or geographical origin [40]. Moreover, distinct clinical manifestations, such as ichthyosis vulgaris and Dennie–Morgan folds, among others, are often associated with specific phenotypic classifications of AD [41].

However, recent research has shifted the focus toward “endotypic” characteristics, such as skin barrier integrity, intracellular lipid abnormalities, and immune pathway dysregulation. These features are increasingly recognized as more effective for stratification, as they provide deeper insights into therapeutic responses [42].

AD exhibits substantial phenotypic and endotype heterogeneity. The extrinsic phenotype (80% of cases) is characterized by elevated serum IgE levels, filaggrin mutations, and a predominant Th2 immune response (IL-4, IL-13, IL-5). In contrast, the intrinsic phenotype (20%) presents with normal IgE levels, increased involvement of the Th1, Th17, and Th22 pathways (IFN-γ, IL-17, IL-22), and greater resistance to therapies typically targeting the Th2 axis [40,43].

In this context, the relationship between phenotype and endotype has a direct impact on therapeutic choices. For example, biologic drugs targeting the IL-4/IL-13 axis (dupilumab, tralokinumab, lebrikizumab) are mainly effective in patients with Th2-dominant inflammation. In cases with Th1/Th17/Th22 activity, which is typical for intrinsic AD, adults, or patients of Asian descent, JAK inhibitors (e.g., baricitinib, upadacitinib) demonstrate greater efficacy [44]. Moreover, clinical features may suggest underlying immunologic mechanisms; for instance, palmar and plantar hyperlinearity indicates a Th2-type barrier defect, whereas psoriasis-like lesions and pronounced lichenification in Asian patients correlate with Th17 predominance [43]. Furthermore, the integration of phenotype and endotype using biomarkers (e.g., TARC, IL-13, IL-22) enables the precise selection of therapeutic options. For example, a high eosinophil profile, associated with IL-5 and disease severity, may indicate the need for targeted therapy with broader immunologic action. At the same time, the lack of correlation between circulating cytokines and skin status suggests the need to analyze both blood and skin biopsy samples [45].

Therefore, treatment selection should be based not only on clinical presentation but also on the combination of phenotypic features, immunologic mechanisms, and biomarker profiles. This approach increases therapeutic efficacy and reduces the risk of treatment failure.

Predictive biomarkers have emerged as crucial tools for tailoring treatment approaches. For example, elevated serum levels of periostin and DPP-4 have been identified as significant markers for predicting favorable responses to anti-IL-13 therapies, such as tralokinumab [36]. Clinical studies have shown that a high level of periostin in serum is correlated with an increased clinical response to therapy with this monoclonal antibody, which makes this biomarker useful in identifying patients with a dominant Th2-type response [20].

Similarly, high tissue levels of IL-22 have been linked to responsiveness to fezakinumab, an IL-22 inhibitor [18]. This cytokine is involved in epidermal hyperplasia and the dysfunction of the skin barrier. High levels of IL-22 in the skin of patients correlated with clinical improvement in response to therapy, suggesting its utility, particularly in patients with lichenified forms of AD in whom IL-22 expression is elevated. Such inflammatory profiles are characteristic of adult patients and those with a chronic disease course in whom a Th22-type response predominates [20,36].

DPP-4, an enzyme involved in T-cell activation, has also been identified as a potential predictive biomarker of the response to tralokinumab therapy. High DPP-4 concentrations were detected in patients showing a favorable treatment response, indicating its usefulness in selecting candidates for IL-13–targeted therapies [20,43].

All three biomarkers (periostin, IL-22, and DPP-4) hold particular clinical relevance in patients with severe forms of AD in whom conventional immunosuppressive treatments are either ineffective or poorly tolerated. This is especially applicable to patients with specific disease endotypes, such as those with a predominant Th2 response (in whom periostin and DPP-4 expression may be elevated) or those presenting with chronic lichenified lesions (associated with high IL-22 levels). Ethnic and age-related differences also influence the utility of individual biomarkers, as the higher Th22 and Th17 pathway activity observed in adults and Asian patients may indicate a greater role for IL-22 in these populations [43,46].

Nevertheless, no clinically approved treatment algorithms for AD currently incorporate biomarkers, such as IL-13, IL-22, CCL17, or FLG mutations. Although these biomarkers have been shown to correlate with disease severity, treatment response, and the risk of atopic comorbidities, they are not employed in therapeutic decision-making [20,47]. In the ECZTRA and ECZTEND trials, the effects of tralokinumab, an IL-13 inhibitor, were evaluated in patients with moderate to severe AD. The treatment resulted in reductions in IL-13, IL-22, TARC, and DPP-4 levels, as well as the restoration of barrier-associated proteins such as filaggrin and loricrin, and improvements in skin microbiota composition. However, baseline biomarker profiles neither guided treatment selection nor influenced therapeutic adjustment [20]. In pediatric AD, elevated Th2/Th22 cytokines (IL-13, IL-22), reduced NMF, increased transepidermal water loss, and FLG mutations are associated with more severe disease and a higher risk of comorbid conditions such as asthma and food allergies. These biomarkers offer prognostic value but are not currently utilized to tailor therapeutic strategies [47]. Similarly, in allergic contact dermatitis, markers such as loricrin, IL-37, CD47, IL-31, and TRPV1 have been studied for their diagnostic and therapeutic potential. Despite their established roles in disease pathogenesis, they have not been integrated into clinical treatment algorithms [48,49].

Thus, despite promising data, none of these biomarkers have been validated for routine clinical application. Their incorporation into practice requires further investigation in large, diverse patient populations. Nevertheless, current findings underscore the potential for biomarker-based stratification to enable personalized approaches in AD management [20,47].

In contrast to adults, pediatric AD is characterized by a stronger activation of the Th2 and Th22 responses, which results in elevated levels of cytokines such as IL-4, IL-13, and IL-22 [50]. This heightened activity of the Th2/Th22 axis correlates with the characteristic clinical phenotype in children, namely the frequent involvement of facial skin and generally milder disease course (4). Biomarkers such as IL-13, IL-22, and mutations in the FLG, which contribute to epidermal barrier dysfunction and represent potential therapeutic targets, therefore, play an important role in treatment [36,51].

In elderly patients, late-onset AD is frequently observed—studies indicate a high proportion of individuals diagnosed after the age of 51, and even after the age of 65 [52]. In older adults, the disease tends to show less involvement of the head, neck, and limbs and is less frequently generalized compared to younger patients. Despite comparable levels of skin lesion severity, as measured by the EASI index, clinical presentation differs, with elderly individuals more commonly exhibiting features such as lichenification, xerosis, and a distinct distribution of lesions [53]. Age-related changes in the skin, such as increased transepidermal water loss, reduced ceramide content, and impaired filaggrin expression, may lead to increased susceptibility to allergens and irritants, as well as enhanced Th2-, Th17-, and Th22-mediated inflammatory responses [54]. In terms of treatment, immunosuppressants such as cyclosporine, although effective, exhibit an unfavorable safety profile in geriatric patients, exposing them to a higher risk of adverse effects [54]. An alternative is biological therapy, particularly dupilumab, which has been shown in clinical trials to be effective and safe in individuals over the age of 65, with sustained therapeutic effects for at least 52 weeks [55,56].

Moreover, the presence of comorbidities in patients with AD is of significant importance, with the most common being asthma, hypersensitivities, food allergies, obesity, and certain autoimmune and metabolic disorders [57,58]. As a result, the treatment of AD increasingly requires therapies aimed not only at cutaneous symptoms but also at controlling systemic inflammation. However, in such cases, there is an increased risk of pharmacological interactions and adverse effects [59]. Janus kinase (JAK) inhibitors, which exert broad anti-inflammatory effects, represent a promising therapeutic option for patients with extensive multimorbidity, offering rapid pruritus control and the potential modulation of inflammatory pathways in comorbid conditions [60].

Special attention must also be paid to the treatment of AD during pregnancy. Hormonal modulation of the immune system in pregnancy favors Th2 dominance, which may lead to the exacerbation of AD symptoms. Consequently, a significant proportion of patients experience a worsening of skin lesions during pregnancy [61,62], which is associated with the risk of complications such as eczema herpeticum, premature rupture of membranes, or neonatal staphylococcal sepsis [62]. Systemic pharmacotherapy during this period is subject to considerable limitations—many immunosuppressive drugs, such as methotrexate, mycophenolate mofetil, and alitretinoin, are strictly contraindicated due to their proven teratogenic effects [63]. Cyclosporine A, azathioprine, and the short-term use of prednisolone are considered possible options in selected cases. Non-invasive therapies, such as emollients and UVB phototherapy, with well-documented safety profiles, are recommended as first-line treatment during pregnancy and lactation [64]. Cohort analyses and systematic reviews do not indicate a significant increase in adverse fetal outcomes, such as miscarriage or congenital anomalies, in patients treated with dupilumab [61,62,65]. Nevertheless, the number of available studies and clinical cases is limited, and long-term safety has not yet been clearly established.

## 5. Current Therapies for AD

### 5.1. Biologics

In recent years, biologic drugs have been introduced in the treatment of AD, specifically targeting immunological mechanisms that drive the development of the disease. Among these therapies is dupilumab, the first biologic agent approved by the FDA and EMA for the treatment of moderate to severe AD in adults and children over six months of age [66]. This approval is particularly significant, as AD affects 3% to 40% of children under the age of six [67]. Notably, approximately 50% of cases manifest within the first year of life, and 95% of patients develop the disease before the age of five [68].

Dupilumab is a monoclonal antibody targeting the alpha subunit of the interleukin-4 receptor (IL-4Rα), thereby blocking the signaling pathways of both IL-4 and IL-13 [69]. These cytokines are central to the pathogenesis of AD, as they drive the Th2 inflammatory response and contribute to skin barrier dysfunction [70]. By inhibiting IL-4Rα, dupilumab effectively disrupts the signaling cascades of IL-4 and IL-13, resulting in reduced inflammation, alleviation of pruritus, and restoration of skin barrier function. These effects collectively lead to significant clinical improvements in patients with moderate to severe AD [71]. A pooled analysis of the LIBERTY AD SOLO 1 and SOLO 2 studies further demonstrated the efficacy of dupilumab monotherapy in significantly alleviating symptoms of AD compared with a placebo. This included marked and rapid improvements in clinical severity scores, reductions in anxiety and depression symptoms, and enhancements in health-related quality of life (HRQoL) [72].

Another biologic therapy approved by the EMA is tralokinumab [73], a fully human monoclonal IgG4 antibody that selectively binds to IL-13 [74]. This binding prevents IL-13 from interacting with its receptors, IL-13Rα1 and IL-13Rα2. Consequently, the formation of the IL-13Rα1/IL-4Rα receptor complex, which is critical for IL-13-mediated signaling, is inhibited [75]. By targeting IL-13, tralokinumab enhances skin barrier function and corrects immunological abnormalities, leading to improved microbial diversity within the skin microbiome. This therapy reduces the abundance of S. aureus, a key driver of microbial dysbiosis in AD, while fostering the growth of beneficial coagulase-negative staphylococci [76]. Long-term studies have demonstrated that tralokinumab maintains its efficacy over extended periods, providing sustained improvement in AD symptoms [77]. In the United States, tralokinumab is FDA-approved under the trade name Adbry™, while in the European Union, it is marketed as Adtralza^®^ [78].

In clinical trials, tralokinumab has demonstrated efficacy in alleviating the symptoms of AD, with a safety profile comparable to that of dupilumab. Both therapies, when administered in conjunction with topical corticosteroids (TCSs), exhibit similar efficacy in achieving key clinical endpoints, including Eczema Area and Severity Index-50 (EASI-50), EASI-75, and EASI-90, by week 32 [79].

### 5.2. Janus Kinase Inhibitors

AD is driven by cytokine signaling through the JAK-STAT pathway. The JAK family, comprising JAK1, JAK2, JAK3, and TYK2, plays a crucial role in transmitting signals from cytokine receptors involved in inflammation and pruritus. JAK inhibitors (JAKi), which selectively block these pathways, have emerged as effective treatments for AD [80]. By targeting multiple cytokines, JAKi helps reduce skin inflammation, alleviate itching, and improve skin barrier function. Their rapid onset of action makes them a promising option for breaking the itch-scratch cycle and managing AD more effectively than traditional therapies [81]. The FDA has already approved the oral JAK inhibitors upadacitinib and abrocitinib. Baricitinib, an oral selective JAK1/JAK2 inhibitor, has been approved in many countries for moderate to severe AD in patients who are candidates for systemic therapy [82,83]. The efficacy and safety of baricitinib with background TCSs in patients with moderate to severe AD and inadequate response, intolerance, or contraindication to ciclosporin A were evaluated in a randomized, placebo-controlled, phase 3 clinical trial. This study confirmed that baricitinib at a dosage of 4 mg in conjunction with TCSs improved the signs and symptoms of moderate to severe AD through 52 weeks of treatment. Importantly, all the groups receiving baricitinib (at doses of 1 mg, 2 mg, and 4 mg) maintained statistically substantial improvements compared to the placebo in terms of the percentage change from baseline in EASI score until week 52 [84].

## 6. Emerging Therapies

Therapeutic approaches to AD have advanced significantly in recent years. These include emerging topical agents, systemically administered monoclonal antibodies, and small-molecule inhibitors (Table 1) [81]. Unlike current treatments, many of these new agents target specific immune axes with greater precision, offering differentiated efficacy, onset speed, and safety [81,85,86]. This section provides a synthesis of their mechanisms, trial outcomes, and comparative positioning in clinical care.

### 6.1. Emerging Topical Medications

Topical medications remain foundational in the treatment of AD, especially for patients with mild to moderate disease or those requiring localized therapy. Novel agents in this category target specific immunological and neuroinflammatory pathways implicated in AD, with increasing emphasis on receptor-level modulation to reduce inflammation, itch, and barrier dysfunction. These innovations are especially relevant in pediatric populations and for patients seeking steroid-sparing options [85]. Among them, there are compounds targeting the aryl hydrocarbon receptor (AhR), JAK receptors, transient receptor potential vanilloid subfamily-member-1 (TRPV1), and phosphodiesterase-4 (PDE-4) [81].

AhR mediates epidermal differentiation and is expressed in all types of skin cells. In AD, impaired keratinocyte differentiation and oxidative stress contribute to barrier dysfunction and microbial dysbiosis. Activation of AhR signaling enhances the expression of skin barrier proteins like filaggrin and loricrin, while suppressing proinflammatory cytokines such as IL-4 and IL-13, thereby targeting two key AD mechanisms simultaneously [86]. AhR agonists activate AhR signaling pathways and modulate gene expression, thus downregulating type 2 inflammation and reducing oxidative stress [101]. In May 2022, the FDA approved tapinarof, an AhR agonist, for the therapy of plaque psoriasis in adults [102]. When it comes to AD treatment, in the phase 2b trial, in adolescent and adult AD treated with 0.5% or 1% tapinarof cream, a notable improvement in eczematous lesions and itch was achieved [101]. In a recent phase 3 study, tapinarof demonstrated significant efficacy and favorable safety and tolerability in adults and children down to 2 years of age with AD. Beyond efficacy, tapinarof is notable for its potential as a steroid-sparing agent. Its AhR-mediated anti-inflammatory and barrier-restoring effects make it a viable long-term topical option, especially for patients concerned about corticosteroid side effects. Recent pediatric trials down to age 2 suggest a favorable safety profile across age groups. In a phase 3 randomized trial, tapinarof 1% cream applied daily resulted in a 57.6% reduction in EASI scores at week 8, compared to 28.4% with the vehicle (*p* < 0.001) [103].

Delgocitinib ointment, a topical pan-JAK inhibitor, is used as a treatment for patients with AD in Japan. A phase 3 study (number JapicCTI-205412) revealed that delgocitinib ointment is well-tolerated and significantly effective for up to 52 weeks when applied to Japanese infants with AD [104]. In chronic hand eczema phase 3 trials, delgocitinib cream demonstrated significantly better efficacy compared to the cream vehicle and was well tolerated over a 16-week period. These findings highlight its potential as an effective treatment option for patients with moderate to severe chronic hand eczema [105]. Delgocitinib’s broad JAK inhibition targets multiple cytokines involved in AD (IL-4, IL-13, IL-22, IL-31), making it a uniquely comprehensive topical option [81]. It demonstrated long-term efficacy and tolerability in infants and children, positioning it as an important candidate in pediatric management and for patients requiring chronic topical immunomodulation without steroids, as a 52-week pediatric phase 3 study (JapicCTI-205412) demonstrated EASI-75 achievement in 68% vs. 25% in vehicle (*p* < 0.0001). Long-term safety was favorable, with no treatment-related SAEs and a <3% discontinuation rate [104]. A selective JAK-1 and TYK-2 inhibitor, brepocitinib, proved to be effective and well-tolerated in patients with mild to moderate AD in phase 2b trials [106].

TRPV1 activation releases central neuropeptides, such as substance P, thus mediating histamine-dependent and histamine-independent itch. TRPV1 is upregulated in lesional AD skin and linked to both histaminergic and non-histaminergic itch pathways. Blocking TRPV1 directly interrupts sensory neuron hyperactivation, a mechanism not addressed by traditional anti-inflammatory agents. Asivatrep, a selective antagonist of TRPV1, improved the clinical signs and symptoms of AD and was well tolerated in a phase 3 randomized, vehicle-controlled study with patients aged above 12 years with mild to moderate AD [107]. PDE-4 is responsible for the degradation of cyclic adenosine monophosphate (cAMP) and influences both epithelial integrity and inflammation. In AD, the overactivation of PDE-4 leads to excessive cytokine production (e.g., IL-4, IL-5, IL-31), contributing to inflammation and itch. Inhibiting PDE-4 restores cAMP signaling, suppresses NFκB activation, and promotes skin barrier repair—a mechanistic rationale behind the clinical success of crisaborole and roflumilast [108]. When the activity of the enzyme is inhibited, the cellular level of cAMP increases and causes the downregulation of NFκB, which constitutes a regulator of cytokine production [86]. Among PDE-4 inhibitors, crisaborole is FDA-approved for topical treatment of mild to moderate AD in adult and pediatric patients 3 months of age and older. In contrast, roflumilast is FDA-approved for the treatment of mild to moderate AD in patients aged 6 years and older [85,109]. Another PDE-4 inhibitor, difamilast, has been reported to improve signs and symptoms of AD in adult and pediatric patients in phase 3 clinical trials and has been approved in Japan [110]. The molecular mechanism underlying its activity is based on the suppression of IL-4 production by basophils in skin lesions [110].

Taken together, these topical agents offer new avenues for managing AD by targeting molecular mechanisms more precisely than traditional treatments. Their emerging roles in pediatric use and as part of steroid-sparing regimens position them as essential tools for individualized, long-term management of AD.

### 6.2. Innovative Monoclonal Antibodies

#### 6.2.1. IL-4Rα

Stapokibart (CM310) constitutes a novel humanized IgG4 monoclonal antibody against the IL-4Rα. In adults with moderate to severe AD, it was well-tolerated and efficient in a phase 2 trial. In a phase 3 study, the effectiveness and favorable safety of Stapokibart in adults with moderate to severe AD were further confirmed [87]. However, it is worth noting that the described study included only Chinese patients. For this reason, research involving patients from diverse ethnic backgrounds is necessary.

#### 6.2.2. IL-31Rα

Nemolizumab constitutes an important advancement in targeting IL-31, a key cytokine involved in itch, the serum levels of which correlate with the severity of AD [111]. Nemolizumab, an IL-31Rα antagonist, has been approved in Japan for AD-associated itch in patients aged 13 years and older [112]. In phase 2 trials, nemolizumab administered subcutaneously at 60 mg every 4 weeks showed a 66% reduction in pruritus VAS scores (*p* < 0.001) and a 78% reduction in EASI scores (*p* < 0.001) compared with placebo, when used adjunctively with topical treatments. The pruritus improvement was clinically meaningful within 2 weeks and sustained throughout the 12-week trial. The incidence of adverse events was similar to placebo (36% vs. 34%, *p* = NS), with nasopharyngitis (10%) and injection site reactions (5%) being the most commonly reported [88]. According to another study, the maximal efficacy of nemolizumab, visible as rapid and sustained improvements in cutaneous signs of inflammation and pruritus, was observed at 30 mg administered subcutaneously every 4 weeks compared to 10 mg and 30 mg doses and placebo [89]. Clinical trials concerning the pharmacokinetics, safety, and efficacy of nemolizumab in participants with moderate to severe AD are currently ongoing [90].

#### 6.2.3. IL-33

Etokimab, an IL-33 inhibitor, was assessed in a 16-week phase 2b trial (NCT03533751), but it did not meet its primary endpoint of significantly improving EASI scores compared to placebo. As a result, it is no longer being developed as a treatment for AD [91]. Itepekimab (REGN3500), another IL-33 inhibitor, also exhibited a lack of clinical efficacy [92], which indicates that the role of IL-33 in AD pathogenesis might not be significant.

#### 6.2.4. IL-36R

Spesolimab, a novel anti-IL-36 receptor antibody, has proven its efficacy in the treatment of several inflammatory diseases, including pustular psoriasis (GPP). In the context of AD, the results of the NCT03822832 trial suggested that IL-36 might play a limited role in AD pathogenesis, as treatment with spesolimab showed only modest improvements in symptoms compared to placebo [93,94].

#### 6.2.5. OX40

OX-40 constitutes a co-stimulatory molecule involved in T-cell activation, clonal expansion, and production of memory cells [113]. Several monoclonal antibodies aim to modulate the immune response by inhibiting the activity of OX40 or its ligand (OX40L), potentially reducing the inflammation and symptoms associated with AD. Rocatinlimab, a fully human, non-fucosylated IgG1 monoclonal antibody, targets OX40 to inhibit T-cell activation and proliferation. It selectively decreases OX40+ activated T-cells and suppresses clonal T-cells [114]. Currently, it is in phase 3 trials in adults with moderate to severe AD. In the phase 2b randomized controlled trial (NCT03703102), rocatinlimab 300 mg every two weeks led to a 61.1% reduction in EASI scores by week 16 compared to 15.0% in the placebo group (*p* < 0.0001) [107]. Importantly, improvements persisted up to 16 weeks after treatment discontinuation, indicating potential disease modification [95]. At that time, rocatinlimab proved to reduce Th2/Th22 and pruritus-related mediators in proteomic analysis as well as to downregulate Th2, Th1/17, and Th22-related genes through week 52 (following treatment cessation at week 36) [28]. Adverse events were mild to moderate, with pyrexia (17%) and nasopharyngitis (14%) being the most frequent. The study also observed statistically significant reductions in serum biomarkers (IL-13, IL-22; *p* < 0.01) [95].

Amlitelimab, a non-depleting IgG4 human anti-OX40L monoclonal antibody, constitutes another inhibitor of the OX40-OX40L pathway. In contrast to rocatinlimab, it binds to OX40L and, in this way, prevents interaction between OX40 and OX40L [28]. Amlitelimab not only suppresses antigen-presenting T-cell activation but also blocks T-cell-independent antigen pathways. This dual mechanism reduces pro-inflammatory activity by preventing OX40L back signaling, effectively targeting both type 2 and Th1/Th17/Th22-mediated inflammatory responses [96]. In a phase 2a randomized placebo-controlled trial, treatment with amlitelimab was well tolerated and resulted in clinically meaningful improvements in adult patients with moderate to severe AD. Amlitelimab demonstrated EASI reductions of 80.1% (200 mg dose) and 70% (500 mg dose) at week 16 versus 49.5% in placebo (*p* < 0.001). Adverse events occurred in 31% of patients, primarily headaches and mild injection-site reactions, with no significant between-group differences (*p* = 0.76) [97].

Telazorlimab, another OX-40 inhibitor in the pipeline for AD, constitutes a humanized anti-OX40 IgG1 monoclonal antibody. Phase 2a study in patients with moderate to severe AD proved that telazorlimab was well-tolerated and safe when administered in two 10 mg/kg intravenous doses 4 weeks apart. The results also confirm the effectiveness of the treatment, as a reduction of EASI measured more than 50%, and a significant decrease in Th1, Th2, and Th17/Th22 mRNA expression levels was detected [98]. In the 2b phase study, adults with moderate to severe AD were subcutaneously administered either 300 mg of the discussed monoclonal antibody every 2 weeks or 600 mg every 2 weeks, following a loading dose. The treatment was well-tolerated and led to notable clinical improvement [99].

#### 6.2.6. TSLP

Growing evidence highlights the critical role of TSLP in AD pathogenesis. Released by keratinocytes in response to various stimuli, TSLP drives Th2 responses and pruritus via sensory neuron interaction, making it a promising therapeutic target [16]. Tezepelumab (AMG-157/MEDI9929), an IgG2λ human monoclonal antibody, targets circulating TSLP by binding to its receptor, thereby inhibiting downstream inflammatory pathways. The Phase 2a trial for tezepelumab in moderate to severe AD showed a higher EASI-50 response in the treatment group (64.7%) compared to placebo (48.2%), but this difference was not statistically significant. Safety evaluations showed similar adverse events in both groups [16,27]. A phase 2b trial aiming to further assess its efficacy as monotherapy and adjunct therapy (NCT03809663) has ended prematurely [16]. MK-8226 and CSJ 117 are other TSLP-targeting drugs under investigation, although the results in AD remain inconclusive [27]. Further studies are needed to verify their potential.

#### 6.2.7. Comparative Evaluation of Monoclonal Antibodies and JAK Inhibitors

To support clinical decision-making, it is essential to evaluate how emerging biologics and JAK inhibitors differ in terms of mechanism, efficacy, safety, and positioning (Table 2). JAK inhibitors, such as upadacitinib, gusacitinib, and jaktinib, act intracellularly by inhibiting multiple cytokines via the blockade of JAK1/JAK2/TYK2. Their oral route and rapid onset make them particularly suitable for patients requiring fast itch relief. For example, in phase 3 trials, upadacitinib (30 mg daily) achieved EASI-75 in 70% of patients by week 16 compared to 25% in the placebo group (*p* < 0.0001), with significant pruritus reduction within the first week of treatment. Adverse events included acne (15%), nasopharyngitis (12%), and elevated creatine phosphokinase levels [80]. Abrocitinib, another oral JAK1 inhibitor, demonstrated EASI-75 responses in up to 75% of patients receiving 200 mg daily at week 16, compared to 46% (100 mg) and 14% in the placebo group (*p* < 0.001) [91]. Common adverse effects were dose-dependent, with nausea and headache being more frequent at higher doses. Gusacitinib also achieved EASI-75 in 74.3% of patients, showing promise, particularly in cytokine-rich endotypes [89].

In contrast, monoclonal antibodies like dupilumab, tralokinumab, and nemolizumab offer greater selectivity and are associated with lower systemic immunosuppression and fewer infections, albeit with slower onset and injection-related drawbacks. In the trials, dupilumab achieved EASI-75 in 60–70% of patients at week 16 (*p* < 0.0001), with additional benefits in sleep quality and mental health outcomes. Notably, post hoc analysis revealed that 43% of patients experienced no pain or discomfort at week 16 versus 14% in placebo (*p* < 0.0001) [115]. Tralokinumab, an IL-13-specific inhibitor, achieved EASI-75 in 60% of patients at week 16 in ECZTRA trials, with a favorable safety profile (*p* < 0.001). Compared to dupilumab, tralokinumab had lower rates of conjunctivitis but slightly higher pain scores at the injection sites [57,116]. Lebrikizumab, another IL-13 antagonist, demonstrated EASI-75 rates of 59–73% across multiple phase 3 trials, with a safety profile similar to that of tralokinumab and dupilumab [90].

Among pruritus-dominant patients, nemolizumab reduced pruritus by 66% and EASI scores by 78% (both *p* < 0.001) at week 12, showing rapid and targeted antipruritic efficacy, particularly in combination with topical corticosteroids [84,85].

Agents such as rocatinlimab and amlitelimab, targeting the OX40 axis, show promising, durable immune modulation, especially in treatment-refractory patients. Rocatinlimab achieved EASI-75 in 61% of patients at week 16 (*p* < 0.0001), with effects persisting 16 weeks post-treatment. Amlitelimab demonstrated up to 80% EASI reduction (*p* < 0.001), with low immunogenicity and broad immune modulation involving Th1, Th2, and Th22 pathways [107,110].

**Table 2:** Comparison of emerging systemic therapies for AD.

Agent	Mechanism of Action	Efficacy (EASI/Pruritus)	Common Adverse Effects	Clinical Use	Reference
Upadacitinib	JAK1 inhibitor (oral)	EASI-75: 70% (30 mg), 60% (15 mg) at week 16 vs. 25% (placebo); *p* < 0.0001	Acne, infections, elevated lipids	Suitable for patients requiring rapid symptom control and preferring oral therapy	[80]
Gusacitinib	JAK/SYK inhibitor (oral)	EASI-75: up to 74.3%	Nasopharyngitis, headache	Consider in patients with complex inflammatory profiles involving multiple cytokine pathways	[117]
Dupilumab	IL-4Rα antagonist	EASI-75: 60–70% at week 16; Pruritus NRS ≥ 4: 66%; *p* < 0.0001	Conjunctivitis, injection-site reactions	First-line biologic for moderate to severe AD with strong Th2-driven inflammation	[71]
Tralokinumab	IL-13 antagonist	EASI-75: ~60% at week 16; *p* < 0.001	Injection-site reactions, infections of upper respiratory tract	Appropriate for patients with microbiome dysregulation and IL-13-dominant endotypes	[74]
Nemolizumab	IL-31Rα antagonist	Pruritus decreased by 66%, EASI decreased by 78%; *p* < 0.001	Nasopharyngitis, peripheral edema	Best suited for patients with severe, treatment-resistant pruritus	[112]
Rocatinlimab	Anti-OX40 mAb	EASI-75: up to 61% at week 16; *p* < 0.0001	Pyrexia, chills, nausea	Promising for patients with chronic, relapsing AD and T-cell mediated inflammation	[95]
Amlitelimab	Anti-OX40L mAb	EASI decreased by 80.1% (200 mg) at week 16; *p* < 0.001	Mild to moderate	Potential option for broad immune suppression across Th2, Th1/17, and Th22 pathways	[96,97]
Tezepelumab	TSLP blocker	EASI-50: 64.7% vs. 48.2% placebo; *p* = 0.09 (not significant)	Mild AEs	Investigational; may benefit select patients with strong epithelial cytokine signatures	[16,27]
Lebrikizumab	IL-13 antagonist	EASI-75: ~59–73% at week 16; *p* < 0.001	Conjunctivitis, injection-site reactions, headache	Alternative to tralokinumab; may be preferred in patients who respond better to IL-13-specific inhibition	[118]
Abrocitinib	JAK1 inhibitor (oral)	EASI-75 achieved in up to 60 % (100 mg) and 70–75 % (200 mg) by week 16; *p* < 0.001	Nausea, headache, dizziness, acne, upper respiratory infections; dose-dependent increases in GI symptoms and infections	Oral option for moderate to severe AD patients seeking systemic therapy; dosing can be tailored (100 mg vs. 200 mg) based on efficacy vs. tolerability; approved for adolescents ≥12 years and adults	[119]
Baricitinib	JAK1/2 inhibitor (oral)	EASI-75: 30% (2 mg), 13% (1 mg) at week 16 vs. 8% placebo; *p* < 0.05	Headache, elevated creatine phosphokinase, infections	Consider patients who prefer oral therapy or are unresponsive to topical agents	[120]

Based on current data, biologics such as dupilumab and tralokinumab remain the first-line options due to favorable long-term safety and targeted efficacy. JAK inhibitors may be considered when rapid symptom control is required or when injectable therapies are not feasible. Experimental agents targeting OX40, IL-31, and TSLP offer hope for refractory AD and symptom-dominant phenotypes. However, further long-term head-to-head comparisons are needed to refine their positioning within clinical algorithms.

### 6.3. Novel Oral Systemic Agents

Gusacitinib (ASN002) constitutes an oral inhibitor of Janus (JAK) and Spleen (SYK) tyrosine kinases, which are involved in AD pathogenesis, as they regulate Th1, Th2, and Th17/Th22 pathways. In patients with moderate to severe AD, gusacitinib was well tolerated and showed promising efficacy and rapid onset at daily doses of 40 mg and 80 mg [117]. ASN002 proved its superiority over the placebo in terms of the proportion of patients achieving EASI-50 and EASI-75 and the change from baseline in pruritus [117]. In a phase 2 study concerning treatment of chronic hand eczema, ASN002 was well-tolerated and showed rapid improvement, as patients receiving 80 mg gusacitinib showed a 69.5% decrease in the modified total lesion-symptom score in comparison to 49.0% for 40 mg and 33.5% for placebo [121]. The efficiency of ivarmacitinib (SHR0302), a highly selective JAK-1 inhibitor that presents a mild JAK2-inhibiting effect, has been examined in a randomized, double-blind, placebo-controlled, multicenter, phase 2 trial in patients with moderate to severe AD. In groups receiving 8 mg, 4 mg, and a placebo, EASI75 was achieved in 74.3%, 51.4%, and 22.9% of participants, respectively. The described results indicate the positive impact of oral administration of SHR0302 in treating moderate to severe AD [122]. Jaktinib hydrochloride, a pan-JAK inhibitor, targets JAK1, JAK2, JAK3, and TYK2. Blocking the JAK-STAT signaling pathways effectively reduces inflammation associated with immune responses triggered by cytokines, such as IL-2, IL-4, IL-6, IL-7, and IL-10 [123]. The completion of a multi-center, randomized, double-blind, placebo-controlled, parallel phase 2 clinical study (NCT04539639) was scheduled for May 2022. However, no publications are yet available [124]. Nevertheless, a multicenter, randomized, double-blind, placebo-controlled phase 3 clinical study of jaktinib hydrochloride tablets in the treatment of adult patients with moderate and severe AD (NCT05526222) is currently ongoing [125].

### 6.4. Potential Future Trends

The treatment landscape for AD is entering a transformative phase, with advancements offering more precise therapeutic strategies based on molecular profiles and individual disease phenotypes. These developments are paving the way for highly personalized treatment options tailored to each patient’s needs. Classifying patients based on their phenotype and endotype, which includes phenotypic characteristics and biomarkers, may help identify individuals with different disease progressions and responses to targeted therapies. This approach could enhance the precision of treatments by tailoring therapies to individual patient profiles. Clinical trials also suggest that combining therapies may become a viable strategy when a single treatment proves insufficient. Biologic medicines are expected to maintain a dominant position in the AD treatment market, while JAK inhibitors provide a valuable oral alternative for many patients. However, more extensive, long-term studies are necessary to evaluate the sustained efficacy and safety of these emerging drugs, especially those targeting newly identified cytokines and receptors. Interestingly, therapeutic approaches based on umbilical cord blood-derived human mesenchymal stem cells (hUCB-MSCs), which secrete epidermal growth factor (EGF), have been developed. It has been demonstrated that EGF secreted by hUCB-MSCs can enhance AD by modulating the inflammatory responses of cells, including keratinocytes, Th2 cells, and mast cells [126]. Nevertheless, gaining a deeper understanding of the intricate pathogenesis of AD will undoubtedly pave the way for identifying novel therapeutic targets and innovative treatments. Importantly, in addition to pharmacological advancements, a multidisciplinary approach is critical for improving HRQoL in AD patients. Educating both patients and their caregivers is crucial in managing the condition effectively.

## 7. Combination Therapies and Biologicals

### 7.1. Topical Therapies in Combination with Biologicals

Current standard topical treatments for AD include TCSs, emollients, and topical calcineurin inhibitors [127]. In cases of inadequate response to first-line treatment therapies, combining biologics with TCSs can lead to improved efficacy. It is expected that, in clinical practice, biologics will often be administered alongside topical therapies [127,128].

Dupilumab has been studied in combination with TCSs. In the phase 3 LIBERTY AD CHRONOS trial, the addition of TCSs to dupilumab led to sustained improvement in AD manifestations across the head, neck, trunk, and upper and lower extremities over 52 weeks. Erythema reduction was observed as early as week 2 in most body areas and by week 4 in the upper extremities [129].

The LIBERTY AD CAFÉ evaluated dupilumab + TCSs in adults with an inadequate response or intolerance to cyclosporine A. By week 16, 85% of the patients achieved at least a 50% reduction in EASI. This combination improved the quality of sleep, pruritus, and anxiety [128,130].

In children aged 6–11 years with severe AD, dupilumab with TCSs demonstrated similar improvements in itch, quality of life, anxiety, depression, and sleep. Adverse events (AEs) more frequent than in the placebo + TCS group included conjunctivitis and injection-site reactions. However, the overall incidence of treatment-emergent adverse events (TEAEs) was lower in the dupilumab + TCS group [101].

Other biologics have shown benefits in combination with TCSs. The ADhere trial demonstrated that lebrikizumab + TCSs improved skin clearance, pruritus, and sleep quality by week 8, with early benefits seen as soon as week 4. TEAEs were predominantly mild to moderate, including conjunctivitis, headache, and injection-site reactions [115]. In the ECZTRA 3 study, tralokinumab combined with TCSs as needed led to sustained clinical improvement over 32 weeks, with early effects on sleep and itch. Notably, the reduced need for TCSs in the active arm supports tralokinumab’s role as a TCS-sparing agent [131].

Nemolizumab, an anti-IL-31 receptor antibody, was evaluated in ARCADIA 1 and 2 trials in combination with background TCS ± TCI. It provided rapid itch relief by week 1 and sleep improvement by week 16. Although the overall safety profile was comparable to placebo, higher rates of facial edema and asthma were observed in the treatment group [132].

Collectively, these studies support the efficacy of combining biologics with TCSs in improving both clinical and patient-reported outcomes (PROs). Such regimens may also reduce dependence on long-term topical corticosteroid use. However, further research is needed to establish the optimal duration of concomitant therapy, clarify safety profiles in long-term use, and assess cost-effectiveness in real-world settings. Additionally, standardization of treatment protocols and outcome reporting would enhance the comparability of future studies.

### 7.2. JAK Inhibitors in Combination with Biologics

Recent real-life data suggest that combination therapy with biologics—tralokinumab and a JAKi (upadacitinib)—may offer clinical benefits in patients with severe, treatment-refractory AD. In a monocentric case series, four patients with recalcitrant AD achieved rapid and significant improvement (Validated Investigator Global Assessment for Atopic Dermatitis score of 0–1 or a ≥75% SCORAD reduction) within a median of 2.5 months after initiating the combination therapy. Although the treatment was discontinued upon reaching therapeutic goals, relapses occurred in all cases. No major adverse events were observed; however, laboratory abnormalities such as dyslipidemia and transaminitis were noted, underscoring the need for monitoring [133]. However, despite promising real-world evidence, there is a lack of large-scale, multi-center studies to confirm these findings; data remain limited, and further research is needed to establish the safety profile, cost-effectiveness, and long-term efficacy of this combination therapy. There is a need for careful monitoring of combination therapy with JAKi and biologics due to the possible increased risk of infection and tuberculosis (TB) reactivation. It has been demonstrated that some JAKi, as well as certain biologics, elevate the risk of TB reactivation [134]. However, dupilumab may potentially reduce the risk of infection, particularly skin infections, possibly by restoring the skin barrier function, which may be beneficial in combination with JAKi. Nevertheless, there are no clear data regarding the infection risk associated with the combination of these therapies.

### 7.3. Dupilumab in Combination with Phototherapy

Although data on the combination of phototherapy and biologic agents in AD remains limited, several reports have examined the concomitant use of dupilumab and narrow-band ultraviolet B (NB-UVB) phototherapy. Given that the clinical efficacy of dupilumab typically emerges after 12–16 weeks, adjunctive therapies are often considered to achieve more rapid symptom control [116]. In a pilot study involving 45 patients, NB-UVB phototherapy was associated with enhanced clinical improvement at week 4 compared to monotherapy; however, this additive effect diminished by weeks 12 and 16. Importantly, the combination was well-tolerated, with no significant adverse events reported. A promising case report involving a pediatric patient demonstrated comparable therapeutic effects [135]. The authors concluded that the combination of dupilumab with NB-UVB phototherapy represents a potentially effective treatment modality, offering accelerated clinical improvement and the possibility of reducing the required doses of both TCS and dupilumab. These findings suggest that NB-UVB phototherapy, when used as a bridging strategy during the initial phase of dupilumab therapy, may offer a safe, well-tolerated, and cost-effective approach to accelerating remission in patients with severe AD.

### 7.4. Dupilumab and Microbiome-Based Therapies

Administration of dupilumab significantly alters cutaneous fungal and bacterial microbiota in patients with AD, leading to a reduction in *Malassezia* colonization and a decreased abundance of *Staphylococcus aureus*. These changes contribute to a skin microbiome composition that more closely resembles that of healthy individuals [136]. In a randomized clinical trial conducted in China, treatment with dupilumab in 27 patients with AD led to significant improvements in tryptophan metabolism, increased gut microbiota diversity, and partial reversal of intestinal dysbiosis [137]. In a meta-analysis of six studies, probiotics significantly reduced disease severity in adults with moderate AD, as indicated by a mean decrease in the SCORAD score [138]. However, no significant effects were observed on skin severity, itch intensity, quality of life (Dermatology Life Quality Index (DLQI), Skindex), or immunological markers, including IL-4, IFN-γ, and serum IgE levels.

Although experimental data on the combined use of biologics and microbiome-based therapies are lacking, this approach may represent a cost-effective treatment modality due to dupilumab’s modulatory effects on the microbiome and the potential adjuvant benefits of microbiome-targeted interventions.

## 8. Challenges and Limitations

The use of biological therapies is associated with significant safety concerns and potential adverse effects. Dupilumab has demonstrated high efficacy in reducing the severity of AD symptoms and improving patients’ overall quality of life. However, adverse effects, such as conjunctivitis and blepharitis have been reported in 22.1% of cases [139,140]. In real-world settings, these complications have been observed in 38.2% of patients [141]. Nevertheless, these adverse effects were transient, and in most cases, continued dupilumab therapy was feasible, allowing for symptom resolution. In a real-world study by de Wijs et al., eye symptoms were reported in 62% of patients treated with dupilumab; however, only five patients discontinued therapy due to adverse events, indicating that treatment was generally well tolerated and manageable in routine clinical practice [142]. It is important to emphasize that the current scientific literature does not clearly elucidate the mechanisms underlying these adverse effects. However, it is most commonly suggested that the blockade of the IL-4/IL-13 pathway may disrupt the homeostasis of the ocular surface [143]. Furthermore, studies have highlighted that the severity of AD itself constitutes a significant risk factor for the aforementioned ocular complications, with patients suffering from more severe forms of the disease being more susceptible to eye-related adverse effects [144].

Dupilumab is one of the few systemic therapies approved for moderate to severe AD in adolescents, with limited real-world data currently available [145].

An important aspect of the safety profile of dupilumab in AD is the potential risk of infection. Although dupilumab is not a classical immunosuppressant, its impact on the immune system raises certain concerns. While the majority of clinical studies have not conclusively demonstrated an increased risk of infections in patients treated with dupilumab compared to placebo, some studies, particularly phase 2b trials, have reported an elevated incidence of herpes infections. Dupilumab significantly improved all pre-specified efficacy endpoints versus placebo (*p* < 0.0001), including clinical severity outcomes, PROs, symptoms of anxiety and depression, and HRQoL, consistent with previously published results. Post hoc analyses showed that among patients reporting baseline pain or discomfort, 43% and 46% of patients receiving dupilumab weekly and every two weeks, respectively, reported no pain or discomfort at week 16, compared to 14% in the placebo group (*p* < 0.0001). Improvements were also noted in Investigator’s Global Assessment (IGA) scores and EASI percentages over time in dupilumab-treated patients, with minimal changes seen in the placebo groups. Dupilumab demonstrated rapid improvement in pruritus within 1–3 days of treatment initiation. Regarding safety, no new signals were observed; however, injection-site reactions and conjunctivitis were more common with dupilumab, while AD exacerbation and non-herpetic skin infections occurred more frequently with placebo [146].

The number of studies on dupilumab remains relatively limited due to its recent introduction, and as a result, its full spectrum of adverse effects may not yet be fully elucidated. It is important to emphasize that the absence of long-term safety data may be a key factor contributing to concerns regarding the safety profile of this drug. Nevertheless, sustained clinical improvement has been observed in long-term studies of up to 260 weeks. In an open-label extension with 2677 patients, 67.5% achieved an IGA score of 0 or 1 at week 260, and 88.9% had at least 75% improvement in EASI. The mean EASI score decreased from 16.39 to 2.75. TEAE rates remained stable or declined, with common events including nasopharyngitis, worsening AD, upper respiratory infections, conjunctivitis, headache, oral herpes, and injection-site reactions. Only 8.4% discontinued due to side effects, supporting dupilumab’s favorable long-term safety and efficacy [147].

While dupilumab is generally well tolerated and effective in the long-term management of AD, concerns have been raised regarding its immunogenicity and the potential impact on sustained treatment response. Anti-drug antibodies (ADAs) have been observed in a small proportion of treated patients, with persistent or high-titer responses occurring infrequently, reported in fewer than 2% and 1% of cases, respectively [148]. In rare instances, high ADA titers have been associated with reduced serum drug levels and diminished clinical efficacy, suggesting that immunogenicity may contribute to treatment attenuation over time. However, the majority of ADA-positive patients maintain a therapeutic response, and antibody titers often decline with continued therapy [149]. Similarly, long-term observational studies, including data from the BioDay registry, have shown that most patients continue to benefit from dupilumab for up to five years, with immunogenicity-related treatment failure remaining uncommon [150].

In contrast, dupilumab and tralokinumab are associated with mild injection site reactions (ISRs) and upper respiratory tract infections, which rarely lead to treatment discontinuation. In a study of 392 patients, 27.5% of dupilumab and 60% of tralokinumab patients reported ISRs. Symptoms included pain, swelling, burning, and erythema, with tralokinumab patients reporting higher pain scores. ISRs usually appeared within two months and resolved with topical corticosteroids. No patients stopped treatment due to ISRs in either group [151].

Although lebrikizumab is mentioned as an effective biological agent for AD, the current section lacks specific details regarding its safety profile or long-term data. Further studies will be necessary to establish its risk–benefit profile clearly in both controlled trials and real-world settings. Upadacitinib, a selective JAK1 inhibitor, is approved for treating severe AD in adolescents and adults at daily doses of 15 or 30 mg. In phase 3 trials, upadacitinib demonstrated superior efficacy compared to both placebo and dupilumab after 16 weeks; real-world studies confirmed these findings, with up to 97.5% of patients achieving EASI 75, 82.1% reaching EASI 90, and 69.2% attaining complete skin clearance (EASI 100) at the same time point [152].

Even the most effective therapy will not yield the expected outcomes without adequate patient adherence. In the context of biological therapies for AD, factors such as the complexity of treatment regimens, including the frequency and mode of drug administration, the potential for adverse effects, and the lack of immediate results, may discourage patients from continued use, ultimately contributing to reduced therapeutic efficacy [153].

Long-term management of AD requires confirmation of sustained efficacy and safety beyond 52 weeks. Observational data indicate that dupilumab maintains clinical benefit for up to 84 weeks, with improvements in EASI, DLQI, and POEM scores and an acceptable safety profile, with ocular adverse events being the most frequently reported [154]. Similar outcomes were reported in the PROSE registry, where reductions in disease severity and improvements in quality of life were sustained over a two-year observation period [149]. In the RELIEVE-AD study, conducted over 30–36 months, more than 80% of patients reported satisfactory disease control and a reduced need for concomitant therapies [155]. Despite these encouraging findings, real-world studies have methodological limitations, including the absence of control groups and reliance on subjective outcome measures. Moreover, most long-term data concern dupilumab; for JAK inhibitors, evidence beyond one year remains limited [152]. Drug survival data from a Dutch prospective single-center registry involving 549 adults with atopic dermatitis demonstrated that approximately 30% of patients discontinued treatment within 18 months. Specifically, 18-month drug survival rates were highest for dupilumab (70.0%) and lowest for baricitinib (20.4%), with intermediate rates for abrocitinib (51.5%), upadacitinib (48.4%), and tralokinumab (39.4%). Despite the extensive use of dupilumab as the predominant first-line therapy (87.2%) and upadacitinib as the most frequent second- and third-line agent, no significant predictors of drug survival were identified. These findings underscore the urgent need for reliable biomarkers to predict long-term treatment response and reduce therapy switching, which occurred in 26.8% of patients [156]. As the therapeutic landscape expands, high-quality longitudinal observational studies are crucial for robustly assessing long-term treatment durability and safety in routine clinical practice.

These concerns have led to increased research on biological therapies aimed at modulating the skin microbiome. Recent studies have highlighted the potential of microbiome-targeted therapies, including probiotics, prebiotics, and microbiome-modulating moisturizers, in the treatment of AD by restoring homeostasis and enhancing skin barrier function [157,158,159]. Biologic therapies such as dupilumab and tralokinumab primarily target the Th2 pathway; however, other immune pathways implicated in AD, including Th1, Th17, and Th22, remain unmodulated by these treatments. Consequently, new biologic agents are being developed to target these additional pathways, broadening therapeutic options for AD management [160].

Despite their proven efficacy, next-generation biological therapies and immune modulation strategies face significant challenges related to availability, cost, and the need for personalized treatment approaches. The heterogeneity of AD underscores the necessity for ongoing research to further elucidate the disease’s pathophysiology and develop more effective, individualized therapeutic strategies. As advancements in the field continue, the integration of these novel treatments into clinical practice will require a careful evaluation of their benefits and limitations to ensure optimal patient outcomes.

## 9. Conclusions

AD, as one of the most common skin conditions, poses numerous economic and social burdens. Recent advances in biological therapies offer new treatment options for patients who have not achieved satisfactory results with previous basic management strategies, such as avoidance of trigger factors, barrier repair, and topical anti-inflammatory therapies. Emerging biological drugs recently approved target a variety of pathways. This is particularly significant in the context of AD, whose complex pathophysiology exacts a comprehensive and multi-faceted therapeutic approach. Modern therapies that focus on blocking specific cytokines, JAK kinase, and co-stimulating proteins enable the personalization of treatment to an individual’s specific molecular endotype, thereby maximizing therapeutic effectiveness and minimizing adverse effects.

However, there are some limitations regarding new therapeutic possibilities. These include a lack of long-term studies on their effectiveness and adverse effects in all age groups, the high cost of therapy, and extended treatment duration before desirable effects can be observed. Combining traditional and biological therapies may be pivotal for the widespread use of targeted medications. This approach can enhance efficacy and solve some challenges associated with the exclusive administration of biological drugs. Despite their limitations and challenges, biological therapies represent a promising transformative approach for the treatment of AD.

## Figures and Tables

**Table 1:** Innovative monoclonal antibodies in AD treatment.

Monoclonal Antibody	Target	Trial Identifier	Reference
Stapokibart (CM310)	IL-4Rα	NCT05265923	[87]
Nemolizumab	IL-31Rα	NCT03985943 NCT03989349 NCT03989206 NCT03921411 NCT04921345	[88,89,90]
Etokimab	IL-33	NCT03533751	[91]
Itepekimab (REGN3500)	IL-33	NCT03738423	[92]
Spesolimab	IL-36R	NCT03822832	[93,94]
Rocatinlimab	OX40	NCT03703102 NCT05398445	[28,95]
Amlitelimab	OX40L	NCT03754309 NCT05131477 NCT05492578	[96,97]
Telazorlimab	OX40	NCT03568162	[98,99]
Tezepelumab (AMG-157/MEDI9929)	TSLP	NCT00757042 NCT02525094 NCT03809663	[16,27,100]
